# Effects of Yeast Culture Supplementation on Milk Yield and Milk Composition in Holstein Dairy Cows: A Meta-Analysis

**DOI:** 10.3390/ani15213065

**Published:** 2025-10-22

**Authors:** Hongyan Xiang, Xusheng Dong, Xueyan Lin, Qiuling Hou, Zhonghua Wang

**Affiliations:** 1 College of Veterinary Medicine, Shandong Agricultural University, Tai'an 271018, China; t909490203@163.com (H.X.);; 2 College of Animal Science and Technology, Shandong Agricultural University, Tai'an 271018, China; linxueyan@sdau.edu.cn

**Keywords:** yeast culture, Holstein cows, meta-analysis, milk yield and composition

## Abstract

Yeast culture (YC) is widely used in dairy production to enhance milk yield and quality, yet effects vary due to differences in products, doses, and trial conditions. This meta-analysis evaluated the impact of YC supplementation on milk yield and composition in lactating Holstein cows, aiming to identify effective yeast culture types, dosages, and duration of use. A systematic search of PubMed, Web of Science, and CNKI for randomized controlled trials (RCTs) from 2000 to 2024 was conducted. Following PICOS criteria, 23 RCTs comprising 32 comparisons and over 3200 cows were included. Data were analyzed using RevMan 5.3 and Stata/MP 15.0 to compute standardized mean differences (SMD) and 95% confidence intervals with random-effects models. Subgroup and sensitivity analyses were performed. Results showed that YC supplementation significantly improved milk yield (SMD = 2.14), fat (SMD = 0.57), protein (SMD = 1.34), and lactose content (SMD = 0.61). Subgroup analysis revealed that supplementation with saccharomyces cerevisiae at a dosage of 10–50 g/d effectively increased milk yield during lactation 42–56 d. In contrast, during the lactation 21–30 d, different dosages of saccharomyces cerevisiae exerted differential effects on milk composition: supplementation at 60–120 g/d contributed to an increase in milk fat content, while supplementation at 10–50 g/d significantly enhanced milk protein level. Furthermore, lactose content was not significantly associated with the feeding period of saccharomyces cerevisiae; however, high-dose (>120 g/d) could significantly increase lactose content. Significant heterogeneity (I^2^ = 70.7–89.6%) was observed, largely due to strain and dose variations. In conclusion, YC effectively enhances milk production and composition, with optimal outcomes depending on yeast type, dose, and duration, providing evidence-based recommendations for targeted supplementation strategies.

## Simple Summary

This meta-analysis evaluates the effects of yeast culture (YC) supplementation on milk production and composition in Holstein dairy cows. Based on 23 randomized controlled trials involving over 3200 cows, YC significantly improved milk yield, fat, protein, and lactose content. Supplementation with saccharomyces cerevisiae at a dosage of 10–50 g/d effectively increased milk yield during lactation 42–56 d. In contrast, during the lactation 21–30 d, different dosages of saccharomyces cerevisiae exerted differential effects on milk composition: supplementation at 60–120 g/d contributed to an increase in milk fat content, while supplementation at 10–50 g/d significantly enhanced milk protein level. Furthermore, lactose content was not significantly associated with the feeding period of saccharomyces cerevisiae; however, high-dose (>120 g/d) could significantly increase lactose content. These findings support tailored YC use to enhance specific milk components in dairy herds.

## 1. Introduction

Sustainable and efficient dairy production depends on optimized feeding and management strategies [1]. Among natural feed additives, yeast culture (YC), produced through anaerobic fermentation of *Saccharomyces cerevisiae* on defined media followed by drying to preserve metabolites, has received increasing attention in dairy nutrition [2]. YC contains both yeast biomass and fermentation byproducts, including peptides, amino acids, nucleotides, organic acids, oligosaccharides, and vitamins [3]. Supplementation with YC has been associated with improvements in milk yield and composition, although responses have varied among studies [4].

Several mechanisms have been proposed to explain the observed production responses. YC has been shown to improve organic matter digestibility [5], stabilize ruminal pH [6], and shift microbial populations toward beneficial fermentative species while limiting colonization by undesirable bacteria [7]. Although yeasts are obligate aerobes and do not proliferate in the anaerobic rumen, both viable and inactivated cells can exert metabolic effects [8]. Reported benefits include enhanced microbial nitrogen synthesis [9], improved fiber digestibility and energy utilization, increased volatile fatty acid (VFA) production, and reductions in methane output [10]. In addition, YC has been linked to immune modulation and stress resilience, which may contribute indirectly to improvements in milk yield and composition, including fat, protein, and lactose content [11].

Nevertheless, there remains considerable controversy regarding the efficacy of YC, with its effects likely dependent on dosage, type, and feeding method. The effectiveness of YC appears to depend on strain selection, supplementation level, and feeding duration, yet these factors remain poorly standardized across studies. Some studies have found that the use of YC merely increases milk yield, while other research has identified further functional benefits associated with YC. The current research controversy may stem from small sample sizes and often limited to individual farms or herds, reducing statistical power and limiting the generalizability of results [12]. However, in dairy production, High variability in basal diets, environmental conditions, and YC product characteristics further complicates comparison across studies. Differences between yeast species, such as *S. cerevisiae* and Candida utilis, have received limited investigation, and dose–response relationships have not been systematically defined. These issues have hindered the development of evidence-based YC supplementation protocols for commercial use. While qualitative reviews have summarized trends, they fall short in quantifying effect sizes or accounting for study heterogeneity.

In order to evaluate the application of YC in production more effectively, and to determine the most appropriate YC strain, dosage and feeding duration, as well as their respective effects on milk yield and quality, some researchers have opted to address this issue through meta-analysis. By means of meta-analysis, the results from multiple independent studies are combined. It increases overall sample size, reduces uncertainty, and identifies sources of heterogeneity [13]. This approach is now widely used in animal science to evaluate feed additives and management practices. However, earlier meta-analyses of YC in dairy cattle have included studies with variable design quality, including conference abstracts and industry reports with limited methodological transparency [14,15]. Reviews published before 2012 were based on a small number of trials and lacked the power to detect consistent treatment effects [16]. Moreover, there is a lack of meta-analyses of literature published within the last five years. To address these limitations, this study applies a meta-analytic approach to evaluate three key aspects of YC supplementation in dairy cows. Impact of yeast species, dosage, and feeding duration of yeast culture: A systematic analysis integrating data from multiple independent trials. This study aims to improve statistical resolution and provide a quantitative assessment of YC’s impact on milk yield and major milk components. Standardized mean differences (SMD) and risk ratios (RR), with associated confidence intervals, are used to characterize treatment effects. To examine study heterogeneity, assess publication bias using funnel plots, and conduct sensitivity analyses to test the robustness of the results. Together, these analyses support the development of a more consistent and evidence-based framework for YC use in dairy feeding programs and highlight areas for future research, including long-term production effects and environmental implications.

## 2. Materials and Methods

### 2.1. Search Strategy

A comprehensive literature search was conducted using PubMed, Web of Science, China National Knowledge Infrastructure (CNKI), Wipro, and Wanfang databases for studies published between January 2000 and March 2025. The search combined Medical Subject Headings (MeSH) with free-text terms such as “production performance”, “average daily milk yield”, “feed-to-milk ratio”, “milk composition”, “yeast”, “yeast hydrolysate”, “dairy cow”, and “cattle”. Reference lists of eligible articles were also reviewed to identify additional studies.

### 2.2. Inclusion and Exclusion Criteria

Studies were included if they met the following criteria: (1) randomized controlled trial (RCT) design, (2) Holstein dairy cows as the study population, (3) YC used as the sole dietary intervention, and (4) reported outcomes on milk yield, milk fat percentage, milk protein percentage, or lactose percentage. Only studies involving healthy animals and reporting complete statistical data (means ± SD or SEM) were considered.

Exclusion criteria were: non-RCT designs; unclear outcome definitions or statistical inconsistencies; use of other feed additives in combination with yeast culture; incomplete or unextractable data; use of diseased animals; or high risk of bias (Category C) based on quality assessment.

### 2.3. Literature Screening and Data Extraction

Two independent reviewers conducted a systematic search of Chinese and English databases using predefined Boolean search terms. Titles and abstracts were screened against the eligibility criteria, followed by full-text assessment of potentially relevant studies. Disagreements were resolved through discussion and unresolved cases were referred to a third reviewer.

A standardized data extraction form was used to record the following variables: corresponding author, year of publication, country, parity and lactation stage of Holstein cows, sample size and unit of analysis, duration of intervention, yeast strain and fermentation substrate, complete dietary composition including forage nutrient analyses, milk production outcomes, and measures of variability reported as standard deviations or standard errors.

### 2.4. Quality Assessment

Study quality was evaluated using the revised Cochrane Risk of Bias (RoB 2.0) tool, covering five domains: sequence generation, allocation concealment, blinding, completeness of outcome data, and selective reporting. Studies were classified as high quality if all domains were rated low risk, moderate quality if one or more domains raised some concern, and low quality if any domain was judged high risk. Studies rated as low quality were excluded from the primary analysis.

### 2.5. Statistical Analysis

Meta-analysis was conducted using Stata 14.0 (StataCorp LP) and Review Manager 5.3 (The Cochrane Collaboration). Heterogeneity was assessed using Cochran’s Q test (significance threshold: *p* < 0.10) and quantified using $I^{2}$ statistics [17]. The $I^{2}$ statistic quantifies the proportion of total variability in effect estimates that is attributable to heterogeneity rather than sampling error. It was calculated using the formula:

$$I^{2}=[\frac{Q-\left(k-1\right)}{Q}]\times 100$$

where Q is the Cochran’s heterogeneity statistic and (k − 1) is the degree of freedom, with k denoting the number of included studies. $I^{2}$ values below 25% were considered low, 25% to 50% moderate, and above 75% high.

Given the expected heterogeneity across trials in dairy nutrition, the DerSimonian–Laird random-effects model was applied for primary analysis [13]. This method accounts for both within-study sampling error and between-study variability. A random-effects model was selected based on evidence of between-study heterogeneity ($I^{2}$ > 50% and Q test *p* < 0.10).

Standardized mean difference (SMD) was used as the effect size metric for all continuous outcomes. Calculations incorporated treatment and control group means (μ1, μ2), standard deviations (SD1, SD2), and sample sizes (n1, n2), with pooled standard deviation (SP) used to standardize estimates. Weighted means were calculated using the inverse variance methods, and 95% confidence intervals were generated.

$$SMD=\frac{\mu 1-\mu 2}{SP};\;SP=\sqrt{\frac{\left(n_{1}-1\right)SD_{1}^{2}+(n_{2}-1)SD_{2}^{2}}{n_{1}+n_{2}-2}}$$

A random-effects model was implemented to account for the inherent heterogeneity among dairy cattle feeding trials [13]. This approach was selected over fixed-effects models due to its more conservative estimation properties through incorporation of between-study variance. Consistent with established convention, *Y_i_* represented the effect size estimate for the ith study, and *SE_i_* denoted its standard error [18].

$$\mathrm{Generic}\;\mathrm{inverse}-\mathrm{variance}\;\mathrm{weighted}\;\mathrm{average}=\frac{\Sigma Y_{i}(1/SE_{i}^{2})}{\Sigma (1/SE_{i}^{2})}$$

To explore potential effect modifiers, stratified analyses of yeast culture supplementation was conducted. First, formulations were classified as basal fermentation products, *S. cerevisiae* based preparations, composite cultures, or specialized supplements. Second, dosage-response relationships across the full supplementation range were evaluated. Third, intervention durations were analyzed to assess temporal efficacy patterns. This approach identified key moderators of yeast culture performance while maintaining methodological rigor.

Publication bias was assessed through visual inspection of funnel plot symmetry and tested using Egger’s weighted regression method [19]. Sensitivity analysis was conducted using the leave-one-out method via Stata’s metainf command, sequentially removing individual studies to examine their influence on overall estimates. To further assess sources of variation, meta-regression was performed using SMD as the outcome variable, following established methods for livestock research [20]. When applicable, adjustments were made for clustering effects when the experimental unit differed from the observational unit.

## 3. Results

### 3.1. Selection Process

**Figure 1:**
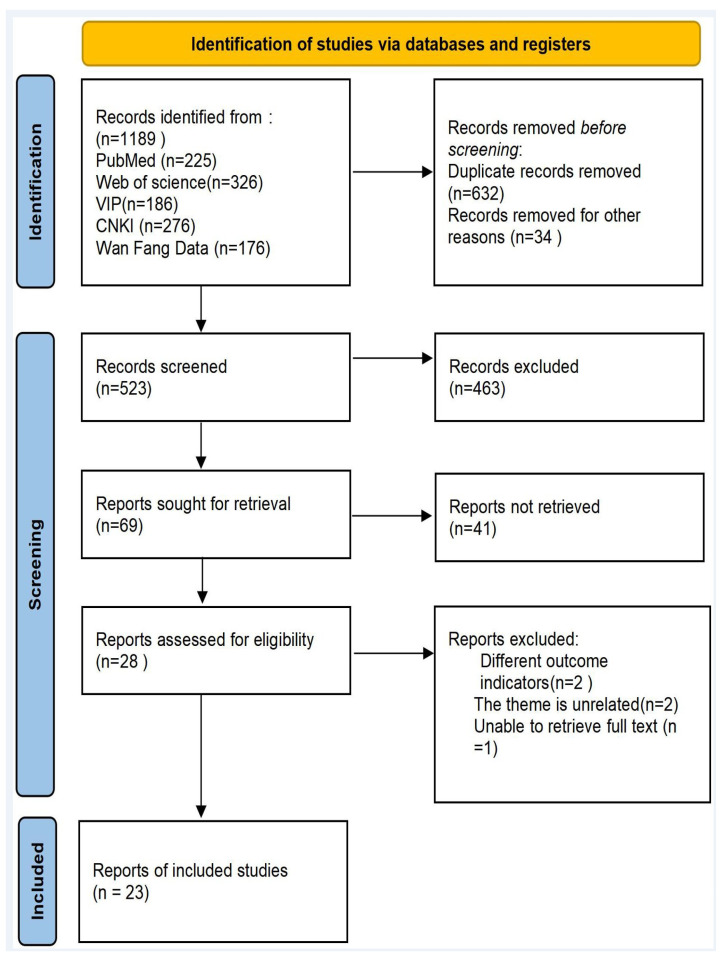
PRISMA flow diagram documenting the systematic study selection process.

The comprehensive literature search identified 1189 potentially relevant articles from five major databases: PubMed, Web of Science, China National Knowledge Infrastructure (CNKI), Wipro Database, and Wanfang Data. After eliminating 632 duplicate records, 557 unique publications were evaluated through title and abstract screening. This initial assessment excluded 463 irrelevant articles. Full-text assessment yielded 23 eligible randomized controlled trials (RCTs) for final inclusion (Figure 1). The selection process followed PRISMA guidelines.

### 3.2. Study Characteristics

Table 1 presents the key characteristics of the 23 RCTs included in this meta-analysis. The compiled studies represent data from over 3200 cow observations when accounting for repeated measurements across trials. Each study is described by its reference information including lead author surname and publication year. The yeast culture products evaluated were systematically classified into four categories: base fermentation products, *S. cerevisiae* based preparations, composite multi-strain formulations, and specialized bioactive supplements.

For each trial, the total number of animals, specific yeast culture product administered, intervention details including daily supplementation dose (ranging from 10 to 50 g/day) and duration (21 to 150 days in milk), along with all measured outcome parameters has been reported. The primary outcomes of interest were milk yield expressed in kilograms per day, and milk composition parameters including fat percentage, protein percentage, and lactose percentage, all reported on a percentage basis.

### 3.3. Quality Assessment

The methodological quality of the 23 included RCTs was assessed using the Cochrane Risk of Bias Tool across six domains: random sequence generation, allocation concealment, blinding of participants and personnel, blinding of outcome assessment, incomplete outcome data, and selective reporting. Risk levels were categorized as low (green), unclear (yellow), or high (red) for each domain.

Computer-generated randomization sequences were properly implemented in 21 studies, while adequate allocation concealment methods were reported in 11 studies. Blinding procedures were well documented, with participant blinding maintained in 22 studies and outcome assessor blinding implemented in all 23 studies. Eight studies reported cases of animal attrition with appropriate handling of incomplete data. No evidence of selective reporting bias was identified in any study.

**Figure 2:**
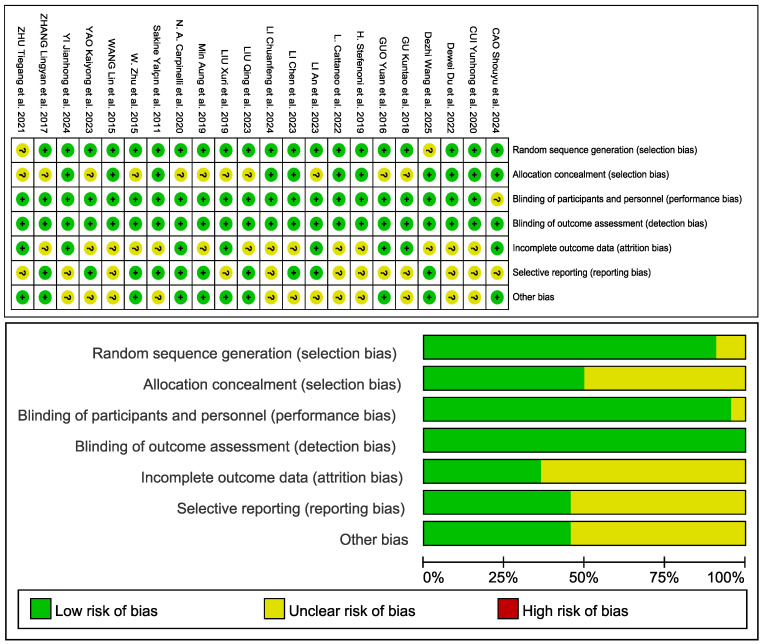
Risk of bias assessment for studies examining yeast culture effects on dairy cow performance [11,21,22,23,24,25,26,27,28,29,30,31,32,33,34,35,36,37,38,39,40,41,42]. "?" indicates an unclear risk of bias; "+" indicates multiple comparison groups from the same study. No red (high risk) markers are shown in the figure, as the risk of bias for all included studies was assessed as either low or unclear.

The overall assessment indicated moderately high methodological quality across the evidence base. The principal limitations involved inconsistent reporting of allocation concealment methods, which were adequately described in only 11 studies, and incomplete documentation of animal attrition in some trials. As presented in Figure 2, most studies demonstrated a low risk of bias across all evaluated domains, supporting the reliability of the synthesized findings.

Evaluation domains include random sequence generation (21 studies low risk), allocation concealment (11 low risk), participant blinding (22 low risk), outcome assessor blinding (23 low risk), incomplete outcome data (8 low risk), and selective reporting (all low risk). The summary evaluation indicates moderately high quality with some concerns regarding allocation concealment and attrition documentation.

### 3.4. Effects of YC on Milk Yield

**Figure 3:**
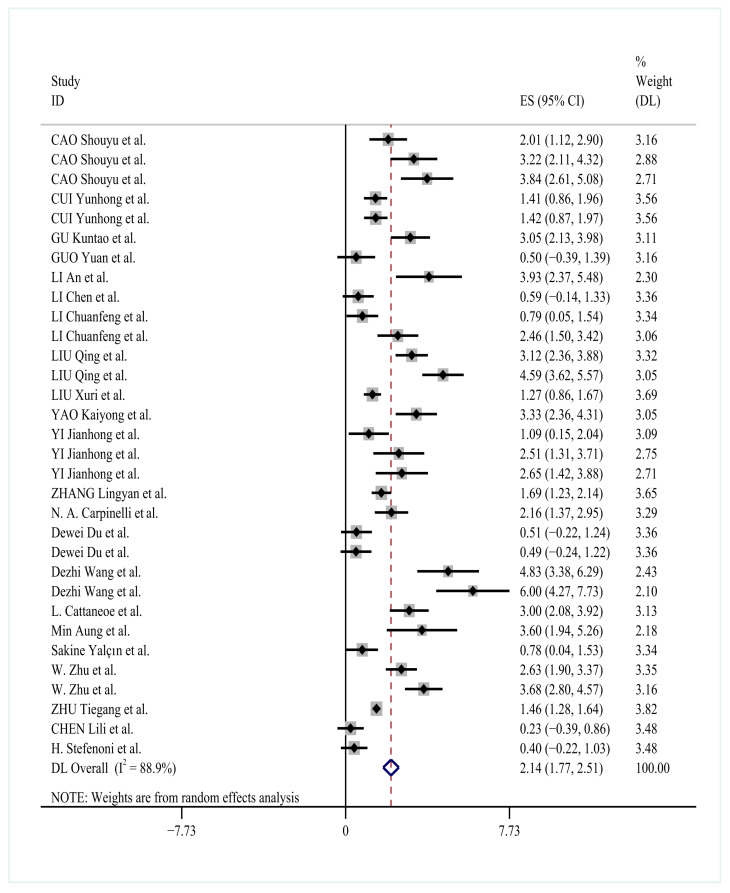
Forest plot of YC supplementation effects on milk yield in dairy cows [11,21,22,23,24,25,26,27,28,29,30,31,33,34,35,38,40,41].

The meta-analysis of 21 studies (32 comparisons, *n* = 3200 cows) revealed significant improvement in milk yield with yeast culture supplementation (SMD = 2.14, 95% CI: 1.77–2.51, *p* < 0.0001) under random-effects modeling (*I*^2^ = 88.9%). Figure 3 presents the forest plot of these results, showing consistent positive effects across studies despite substantial heterogeneity.

Individual study estimates are represented by horizontal lines with proportional weighting squares, while the pooled estimate (SMD = 2.14; 95% CI: 1.77–2.51; *p* < 0.0001) appears as a diamond marker. A dashed vertical line at SMD = 0 provides null reference. The rightward distribution of effect sizes indicates consistent milk yield improvements with yeast culture supplementation. Considerable heterogeneity was observed (*I*^2^ = 88.9%), with subgroup analyses revealing particularly strong effects for basal yeast formulations administered at 10–50 g/d for 42–56 days (SMD = 2.03; 95% CI: 1.83–2.22).

Subgroup analyses identified optimal supplementation parameters for milk yield enhancement. Basal yeast cultures administered at 10–50 g/day for 42–56 days showed the most pronounced effect (SMD = 2.03, 95% CI:1.83–2.22, *p* < 0.0001), as detailed in Table 2. Other yeast types showed comparable but slightly reduced efficacy (SMD range: 1.73–1.87). The persistent high heterogeneity across subgroups (*I*^2^ = 72.4–94.0%) suggests that unmeasured factors, including strain-specific characteristics and non-linear dose–response relationships, may contribute to outcome variation.

### 3.5. Effects of YC on Milk Fat Percentage

**Figure 4:**
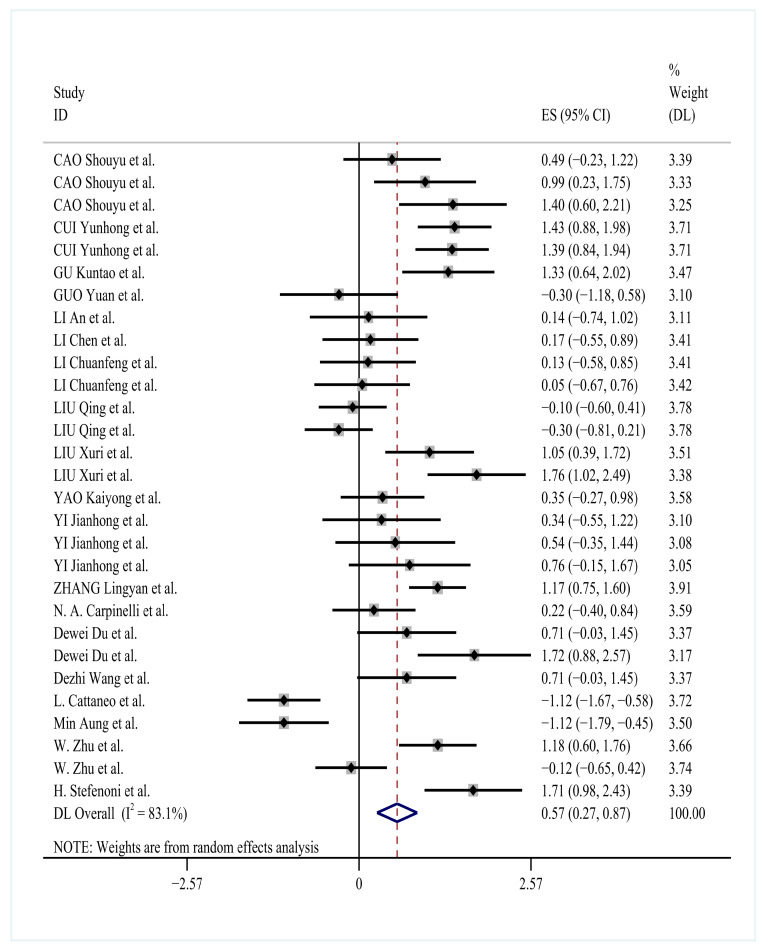
Forest plot showing the effect of YC supplementation on milk fat percentage in Holstein dairy cows [11,21,22,23,24,25,26,27,28,30,31,32,33,34,35,36,38,40,42].

The effect of YC supplementation on milk fat percentage was evaluated using 30 independent comparisons. Pooled analysis under a random-effects model showed a significant positive effect (SMD = 0.57; 95% CI: 0.27 to 0.87; *p* < 0.0001), with substantial heterogeneity among studies (*I*^2^ = 83.1%) (Figure 4).

The SMD was 0.57 (95% CI: 0.27 to 0.87; *p* < 0.0001), indicating a significant positive effect of YC on milk fat percentage.

To explore potential sources of this variation, subgroup analyses were conducted based on yeast type, additive dose, and feeding duration. Heterogeneity decreased notably in the general YC subgroup (*I*^2^ = 67.9%) and in the 60–120 g/d dosage group (*I*^2^ = 54.0%), suggesting that product type and supplementation rate contributed meaningfully to between-study differences.

Among yeast types, general YC were associated with a moderate and consistent improvement in milk fat percentage (SMD = 0.53; 95% CI: 0.37 to 0.69; *p* < 0.0001). *Saccharomyces cerevisiae*-based products produced a slightly higher effect size (SMD = 0.63; 95% CI: 0.40 to 0.85; *p* < 0.0001) but with higher heterogeneity (*I*^2^ = 91.7%). Specialized yeast-based components also showed significant improvement (SMD = 0.57; 95% CI: 0.16 to 0.97; *p* = 0.006), although based on a smaller sample of studies. In contrast, the single comparison involving composite YC showed no significant effect (SMD = −0.08; 95% CI: –0.70 to 0.54; *p* = 0.806).

Analysis by supplementation dose revealed a dose-dependent trend. The 60–120 g/d group produced the largest effect size (SMD = 1.02; 95% CI: 0.78 to 1.27; *p* < 0.0001) and the lowest heterogeneity, indicating both strong and consistent treatment effects. High-dose supplementation (>120 g/d) also increased milk fat percentage (SMD = 0.40; 95% CI: 0.12 to 0.67; *p* = 0.005), although with higher variability (*I*^2^ = 71.8%). The 10–50 g/d group yielded a smaller effect (SMD = 0.37; 95% CI: 0.21 to 0.54; *p* < 0.0001) and the highest observed heterogeneity (*I*^2^ = 87.4%).

Feeding duration further influenced outcomes. The most pronounced improvement occurred with short-term supplementation (21–30 d), with an effect size of 0.68 (95% CI: 0.49 to 0.87; *p* < 0.0001). Intermediate (42–56 d) and extended (60–90 d) feeding periods produced moderate but consistent effects (SMD = 0.43 and 0.46, respectively; *p* < 0.0001 for both), although heterogeneity remained elevated across duration subgroups (*I*^2^ = 82.8% to 84.9%).

Taken together, these findings suggest that the impact of YC on milk fat percentage is influenced by both formulation characteristics and feeding regimen. General YC and intermediate supplementation levels (60–120 g/d) were associated with greater and more consistent improvements. The detailed subgroup outcomes are presented in Table 3.

Subgroup analyses using random-effects models revealed significant variation in milk fat response according to yeast culture characteristics. Heterogeneity analyses indicated substantial variation across studies, with yeast species (*I*^2^ = 67.9% to 94.8%) and supplementation dosage (*I*^2^ = 54.0% to 87.4%) representing the primary sources of heterogeneity. General yeast cultures demonstrated a moderate positive effect on milk fat percentage (SMD = 0.53, 95% CI: 0.37–0.69, *p* < 0.0001). The most pronounced improvement occurred with dosages of 60–120 g/d (SMD = 1.02, 95% CI: 0.78–1.27, *p* < 0.0001). Feeding duration showed less consistent effects across the examined time periods.

### 3.6. Effect of YC on Milk Protein Percentage

**Figure 5:**
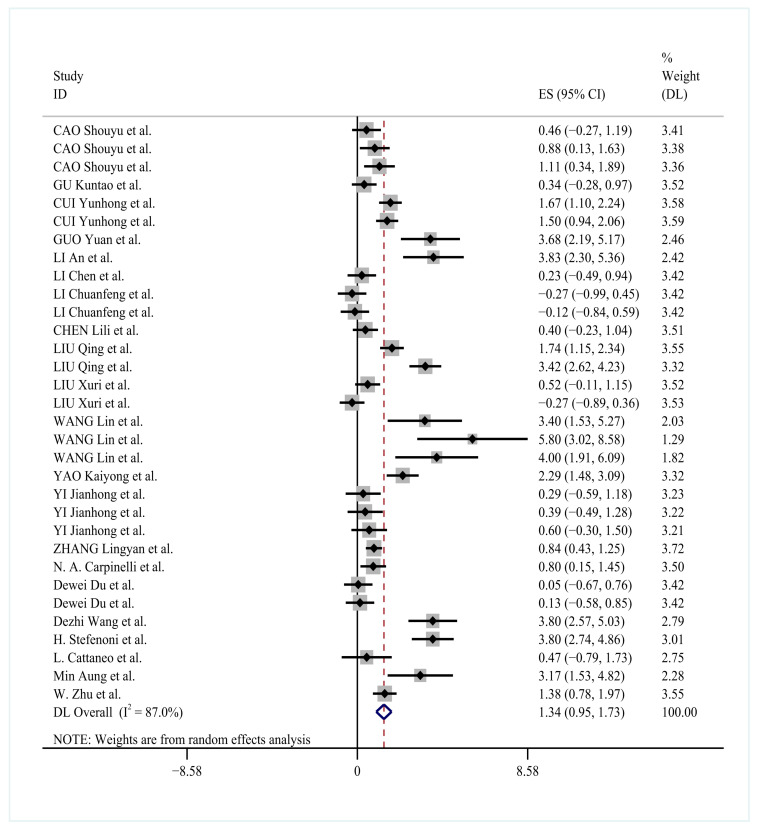
Forest plot of YC supplementation effects on milk protein percentage in dairy cows [11,21,22,23,24,25,26,27,28,30,31,32,33,34,35,36,37,38,40,41,42].

The analysis included 33 effect size estimates from studies evaluating YC supplementation on milk protein percentage in dairy cows. The random-effects meta-analysis revealed significant heterogeneity among studies (*I*^2^ = 87.0%). Yeast culture supplementation increased milk protein percentage compared to controls (ES = 1.34, 95% CI: 0.95–1.73, *p* < 0.0001; Figure 5).

The analysis compares outcomes across YC types (general fermentation products, Saccharomyces cerevisiae-based cultures, specialized bioactive components, and composite formulations), supplementation doses (10–50 g/d, 60–120 g/d, >120 g/d), and feeding durations (21–30 d, 42–56 d, 60–90 d). Random-effects meta-analysis demonstrated significant improvements in milk protein percentage across all subgroups (combined SMD = 1.34, 95% CI: 0.95–1.73; *p* < 0.05). Composite formulations showed the greatest effect (SMD = 1.73), followed by 10–50 g/d supplementation (SMD = 1.15) and 21–30 d feeding duration (SMD = 1.21).

Given the substantial heterogeneity observed, the subgroup analyses of yeast culture type were conducted, supplementation dose, and feeding duration. Composite YC demonstrated the greatest improvement in milk protein percentage (SMD = 1.73, 95% CI: 1.23–2.23), followed by doses of 10–50 g/day (SMD = 1.15) and feeding durations of 21–30 days (SMD = 1.21). The heterogeneity remained high across all subgroups (*I*^2^ range: 87.1–94.3%), with yeast type and dose representing the primary sources of variation (Table 4).

These results were derived from 23 RCTs comprising 32 independent studies (*n* > 3200 Holstein cows). Composite YC administered at 10–50 g/day for 21–30 days yielded the most pronounced improvement in milk protein percentage (SMD = 1.73, 95% CI: 1.23–2.23; *p* < 0.0001). Heterogeneity analysis identified yeast type (*I*^2^ = 87.1–94.3%) and supplementation dose (*I*^2^ = 87.3–88.3%) as primary sources of variation.

### 3.7. Effect of YC on Milk Lactose Percentage

**Figure 6:**
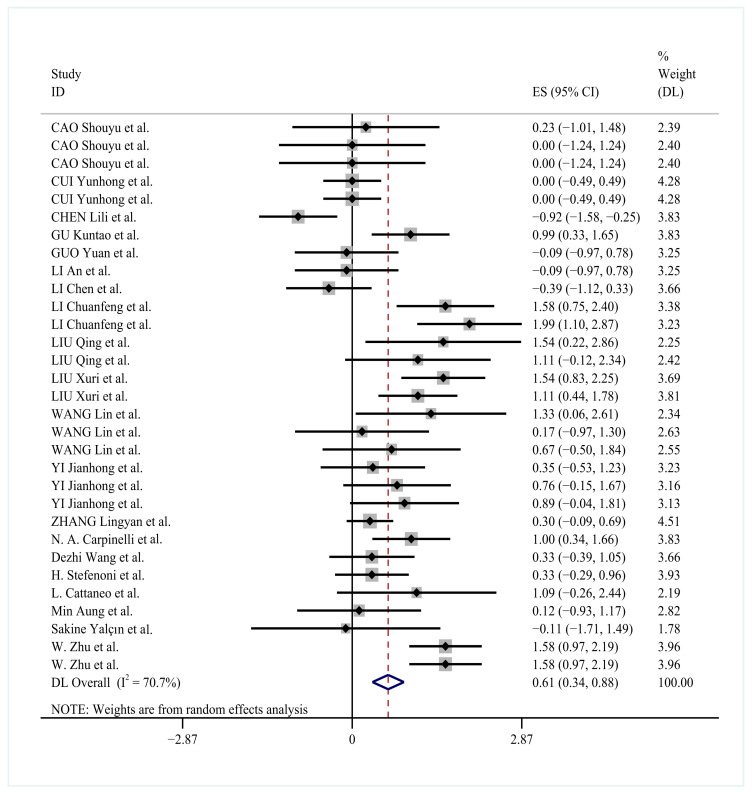
Forest plot of YC supplementation effects on lactose production rate in dairy cows [21,23,25,26,27,30,32,33,34,35,36,37,38,39,40,41,42].

The analysis included thirty-two treatment comparisons evaluating YC supplementation effects on milk lactose percentage. A random-effects model was applied due to substantial heterogeneity (*I*^2^ = 70.7%). YC supplementation significantly increased milk lactose percentage (effect size = 0.61, 95% CI: 0.34 to 0.88, *p* < 0.0001; Figure 6).

The forest plot in Figure 6 presents the combined analysis of 32 independent studies evaluating YC supplementation effects on lactose production in dairy cows. The random-effects model analysis revealed a significant improvement in lactose yield (SMD = 0.61, 95% CI: 0.34 to 0.88, *p* < 0.0001), with substantial between-study heterogeneity (*I*^2^ = 70.7%). Subgroup analyses identified three primary factors contributing to this heterogeneity. Studies utilizing specialized yeast-derived components demonstrated markedly lower heterogeneity (*I*^2^ = 29.0%) compared to other formulations. Similarly, reduced variability was observed in the 10–50 g/d supplementation group (*I*^2^ = 27.0%) and the 21–30 day feeding duration subgroup (*I*^2^ = 44.0%). All subgroup comparisons reached statistical significance (*p* < 0.05).

To investigate the observed heterogeneity, the subgroup analyses of YC type, supplementation duration, and dosage were conducted. Heterogeneity was substantially reduced in subgroups receiving specialized yeast-derived components (*I*^2^ = 29.0%), moderate dosage ranges (10–50 g/d; *I*^2^ = 27.0%), and defined feeding durations (*I*^2^ = 40.5–44.0%). The primary sources of heterogeneity were identified as yeast strain variation, feeding duration differences, and dosage levels. Specifically, Saccharomyces cerevisiae-based cultures, when supplemented at 10–50 g/d for 21–30 days, demonstrated more consistent effects on lactose percentage (Table 5).

The subgroup analyses examined the influence of yeast culture characteristics on lactose production using random-effects models. Heterogeneity analyses revealed substantial variation across studies, with *I*^2^ values ranging from 29.0% to 84.6% for yeast types and 27.0% to 86.4% for dosage levels. The most pronounced improvements in lactose production occurred with specialized yeast-derived components (SMD = 0.56, 95% CI: 0.14–0.98, *p* = 0.008) and the 10–50 g/d dosage group (SMD = 0.43, 95% CI: 0.22–0.65, *p* < 0.0001).

### 3.8. Sensitivity Analysis

To evaluate the robustness of the findings, the sensitivity analyses were conducted by systematically excluding individual studies and recalculating effect estimates. This approach assessed whether any single study disproportionately influenced the overall results for each outcome measure: milk yield, milk fat percentage, milk protein percentage, and lactose percentage.

**Figure 7:**
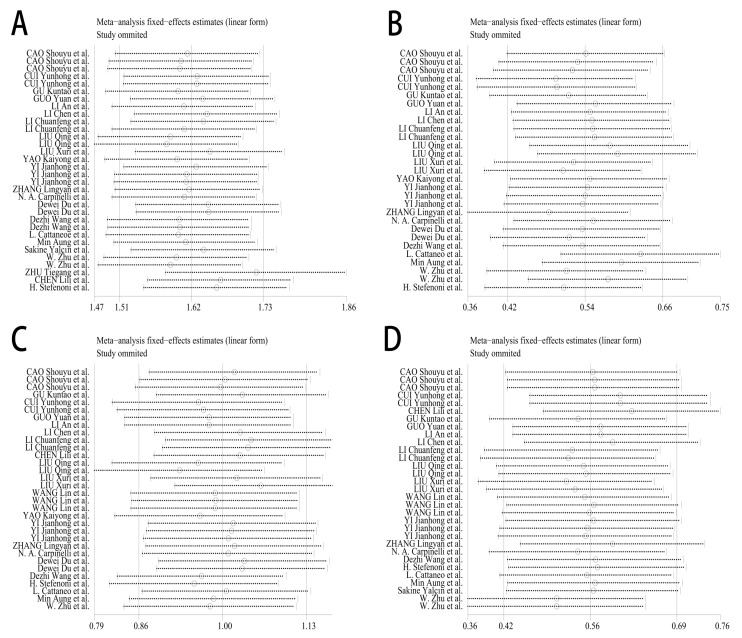
Sensitivity analysis of YC effects. (**A**) Milk Yield, (**B**) Milk Fat Percentage, (**C**) Milk Protein Percentage, (**D**) Milk Lactose Percentage [11,21,22,23,24,25,26,27,28,29,30,31,32,33,34,35,36,37,38,39,40,41,42].

The analysis revealed consistent effect sizes across all exclusions, with no individual study causing significant variation in the pooled estimates for any measured parameter. This consistency demonstrates the statistical stability of the findings and confirms that the meta-analytic results were not unduly influenced by any study. The complete sensitivity analysis results are presented in Figure 7.

The sensitivity analysis evaluated the stability of YC effects on milk production parameters by sequentially excluding individual studies. The pooled effect estimates for milk yield, fat content, protein content, and lactose content remained consistent across all iterations, with 95% confidence intervals showing minimal variation (range: ±0.12–0.18 SMD). This consistency demonstrates the robustness of the meta-analytic findings across different study subsets. The observed stability supports the reliability of these results for informing dairy production decisions regarding YC supplementation.

### 3.9. Publication Bias Assessment

**Figure 8:**
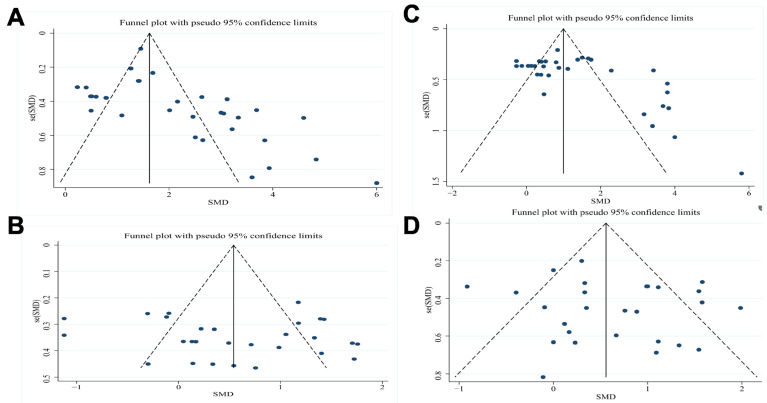
Funnel plot evaluation of publication bias for YC supplementation studies in dairy cattle. (**A**) Milk yield, (**B**) Milk fat percentage, (**C**) Milk protein percentage, (**D**) Milk lactose percentage.

Funnel plot analysis of milk production and composition endpoints demonstrated symmetrical distribution of effect sizes (Figure 8), suggesting minimal publication bias. Egger’s regression test confirmed this observation (*p* > 0.05 for all outcome measures). While these results indicate robust findings, the potential influence of unpublished null results on effect estimates should be acknowledged as a limitation common to meta-analytic approaches.

The symmetrical distribution of effect sizes for milk yield and composition parameters (milk fat, protein, and lactose percentages) indicates low likelihood of substantial publication bias. Egger’s test results (all *p* > 0.05) provide statistical confirmation of this pattern.

## 4. Discussion

This meta-analysis assessed the effects of YC supplementation on milk yield and composition in Holstein cows, drawing on data from 23 RCTs and 32 independent comparisons, involving more than 3200 animals. In addition to calculating pooled effect sizes, sensitivity analyses and Egger’s test were performed to evaluate the reliability of the results and check for publication bias. Due to the clear presence of heterogeneity across studies, a random-effects model was used [43].

Overall, YC supplementation significantly improved production outcomes. Milk yield increased (SMD = 2.14; 95% CI: 1.77–2.51), along with milk fat (SMD = 0.57; 95% CI: 0.27–0.87), protein (SMD = 1.34; 95% CI: 0.95–1.73), and lactose percentages (SMD = 0.61; 95% CI: 0.34–0.88), with all effects statistically significant (*p* < 0.0001). These results support earlier findings that YC can enhance nutrient use efficiency and redirect energy toward lactation by improving rumen function [5].

Variation in study outcomes prompted an exploration of subgroup differences based on yeast type, dosage, and feeding duration. These subgroup analyses not only reduced heterogeneity but also provided clearer insight into how different YC formulations and strategies affect performance.

Several biological mechanisms could explain the observed benefits. YC contains metabolites, such as short-chain fatty acids and nucleotides, that support the growth of key *fiber-digesting bacteria* like *Fibrobacter succinogenes*, improving fiber degradation and volatile fatty acid (VFA) production [44]. It may also help reduce energy losses in the rumen by suppressing *methanogenic microbes*, thereby increasing the energy available for milk synthesis [45]. In addition, β-glucans in YC may influence mammary cell function through immune signaling pathways such as TLR4/NF-κB, enhancing nutrient uptake [46].

Even with these benefits, the response to YC was not consistent across studies. Heterogeneity remained high (*I*^2^ = 70.7–89.6%), and much of it appears to be tied to differences in yeast strains and the non-linear nature of the dose–response relationship. When analyzed separately, trials using standard yeast products at 10–50 g/d for 42–56 days showed the greatest gains in milk yield (SMD = 2.03). In contrast, brewer’s yeast (*Saccharomyces cerevisiae*) used at 60–120 g/d for 21–30 days led to stronger increases in milk fat percentage (SMD = 1.02). These results may reflect differences in metabolite profiles between products. For instance, *S. cerevisiae* is rich in *mannan-oligosaccharides*, which selectively support the growth of butyrate-producing bacteria and enhance acetic acid production for milk fat synthesis [14]. On the other hand, multi-strain yeast blends may boost protein synthesis in the mammary gland by supplying branched-chain amino acids and bioactive peptides such as *glutathione*, potentially activating the mTORC1 pathway [9].

Interestingly, when YC was supplemented at high doses (>120 g/d), lactose percentage continued to rise (SMD = 0.66), but the rate of increase in milk yield slowed (SMD = 1.69 vs. 1.87 at lower doses). This suggests a possible threshold effect, where too much YC might disrupt microbial balance in the rumen. One explanation is that excessive supplementation alters osmotic pressure or suppresses certain microbial groups, such as lactate-utilizing bacteria, that are important for maintaining fermentation stability. Further validation using in vitro fermentation models would help clarify these dose-dependent dynamics [47].

Although sensitivity analyses supported the reliability of the findings, several limitations should be noted. Some studies did not clearly report procedures related to allocation concealment or blinding (Figure 2), which may introduce performance bias and affect the internal validity of those trials. Subgroup analyses were unable to control several biologically relevant covariates, such as parity, forage composition, and environmental conditions including ambient temperature and humidity. For example, responses to YC supplementation may differ between primiparous and multiparous cows due to differences in ruminal development and metabolic priorities, yet stratified data were generally unavailable [14]. Mild publication bias was also suggested by asymmetry in the funnel plot (Figure 8), indicating that studies reporting neutral or negative results may be underrepresented in the published literature. This could result in an overestimation of the overall treatment effect. A further limitation involves the short duration of most trials, which typically did not exceed 90 days. The limited length of these studies prevents assessment of the long-term effects of YC supplementation, including potential microbial adaptation, changes in nutrient partitioning, or sustained alterations in milk composition [8].

Future studies should incorporate high-resolution approaches such as *metagenomics* and *metabolomics* to characterize microbial functional responses to YC. Focus should be given to *ruminal carbohydrate-active enzyme* (CAZyme) clusters and their role in fiber degradation under different supplementation conditions [44]. Establishing causal links between microbial pathways, strain-specific metabolite profiles, and *host phenotypes* will help clarify mechanisms of action. Experimental designs should account for interactive effects of breed, physiological state, and diet, especially differences between Holstein and indigenous dairy breeds at various lactation stages. Studies evaluating combinations of YC with *prebiotics*, such as *inulin*, or *exogenous enzymes* may provide insight into synergistic effects. Dose optimization can be refined using response surface modeling. At the molecular level, further investigation is needed into how yeast-derived metabolites like *nucleotides* regulate mammary gene expression. Integration of mammary *transcriptomics* and single-cell sequencing may clarify their effects on genes such as LALBA and *caseins* [46].

In summary, this meta-analysis confirms that YC supplementation improves milk yield and composition in dairy cows. The findings also highlight how these effects vary with strain type, dose, and duration of feeding. This work supports the development of targeted nutritional strategies aimed at specific production goals, such as enhancing milk protein concentration. It also underlines the need for standardized functional validation of yeast strains and systematic evaluation of dose–response relationships. Incorporating these elements into future studies and industry guidelines will improve the consistency and scientific basis of YC application in dairy nutrition.

## 5. Conclusions

These results suggest that different YC products may be used strategically to target specific production goals. Supplementation with saccharomyces cerevisiae at a dosage of 10–50 g/d effectively increased milk yield during lactation 42–56 d. In contrast, during the lactation 21–30 d, different dosages of saccharomyces cerevisiae exerted differential effects on milk composition: supplementation at 60–120 g/d contributed to an increase in milk fat content, while supplementation at 10–50 g/d significantly enhanced milk protein level. Furthermore, lactose content was not significantly associated with the feeding period of saccharomyces cerevisiae; however, high-dose (>120 g/d) could significantly increase lactose content. This study provides a practical reference for selecting YC types and feeding plans to match herd-level nutritional objectives.

Further work is needed to understand how long-term YC feeding affects production stability, rumen function, and economic returns. Differences in response between Cattle breeds, stages of lactation, and feeding systems should also be studied. Research on how YC affects microbial activity and host gene expression at the cellular level may help improve the precision and consistency of YC use in dairy herds.

## Figures and Tables

**Table 1:** Characteristics of randomized controlled trials examining the effects of yeast culture supplementation on milk production parameters in lactating Holstein cows.

Author	Year	Parity	Days in Milk (days)	Country	Sample Size	Trial Duration (days)	Yeast Culture Type	Treatment Groups	Outcome Measures
CAO Shouyu et al.	2024	-	-	China	20	21	yeast culture	10 g/d, 15 g/d, 20 g/d	①②③④
YAO Kaiyong et al.	2023	2.63 ± 0.21	165 ± 14.2	China	40	56	yeast culture	15 g/d	①②③④
GU Kuntao et al.	2018	2.88 ± 0.05	1–21 Postpartum	China	49	42	yeast β-glucan	10 g/d	①②③④
ZHANG Lingyan et al.	2017	2.04 ± 0.20	136.10 ± 15.80	China	100	30	yeast culture	20 g/d	①②③
YI Jianhong et al.	2024	-	-	China	40	45	yeast culture	50 g/d, 100 g/d, 200 g/d	①②③
LI Chuanfeng et al.	2024	-	-	China	45	56	yeast culture	100 g/d, 200 g/d	①②③④
LIU Qing et al.	2023	2~3	-	China	90	30	yeast culture	25 g/d, 50 g/d	①②③
LI Chen et al.	2021	3.20 ± 0.84	20 ± 3	China	30	90	yeast culture	30 g/d	①②③④
ZHU Tiegang et al.	2021	2.62	116.01	China	600	31	Saccharomyces Cerevisiae Cultures	120 g/d	①
CUI Yunhong et al.	2020	-	28–135	China	64	28	Saccharomyces Cerevisiae Cultures	60 g/d	①②③
LIU Xuri et al.	2019	3.2 ± 0.5	35 ± 5	China	60	56	yeast culture	100 g/d, 200 g/d	①②③④
WANG Lin et al.	2015	2.55 ± 1.24	135 ± 15	China	24	56	compound yeast cultures	80 g/d, 100 g/d, 120 g/d	①②③④
LI An et al.	2023	2.50 ± 0.45	120.10 ± 31.45	China	20	30	compound yeast cultures	12.5 kg/TMR	①②③④
GUO Yuan et al.	2016	-	-	China	20	30	compound yeast cultures	400 g/d	①②③④
CHEN Lili et al.	2019	-	100–160	China	38	30	compound yeast cultures	200 g/d	①②③④
W. Zhu et al.	2015	2.88 ± 0.91	204 ± 46	China	81	56	Saccharomyces Cerevisiae Cultures	120 g/d, 240 g/d	①
Dezhi Wang et al.	2025	2.6 ± 0.14	0–21, 22–60	China	60	60	yeast culture	150 g/d	①②③④
Dewei Du et al.	2022	1.8 ± 0.6	158 ± 14	China	45	60	Saccharomyces Cerevisiae Cultures	30 g/d, 100 g/d	①②④
Min Aung et al.	2019	-	81 ± 7	Japan	32	56	yeast cell wall	10 g/d	①②③④
Sakine Yalçın et al.	2011	-	90 ± 35	Turkey	6	50	Saccharomyces Cerevisiae Cultures	50 g/d	①②③④
L. Cattaneo et al.	2022	2.7 ± 0.7	-	Italy	10	70	Saccharomyces Cerevisiae Cultures	10 g/d	①②③④
H. Stefenoni et al.	2019	2.9 ± 0.2	−21–60	America	40	81	enzymatically hydrolyzed yeast	28 g/d, 56 g/d	①②③④
N. A. Carpinelli et al.	2020	2.62 ± 0.3	−30 ± 6 to 50	America	40	80	yeast culture	114 g/d	①②③④

The control groups for all studies received a basal diet without YC supplementation. Outcome measures are denoted as follows: ① Milk yield, ② Milk fat percentage, ③ Milk protein percentage, ④ Lactose percentage. Specific animal demographics (e.g., parity, precise lactation stage) are not presented due to inconsistent reporting across the primary literature. YC, yeast culture; TMR, total mixed ration [21,22,23,24,25,26,27,28,29,30,31,32,33,34,35,36,37,38,39,40,41,42,43].

**Table 2:** Subgroup meta-analysis by culture type, dosage, and feeding duration on milk yield in dairy cows.

Category	Subgroup	Studies (*n*)	*I*^2^ (%)	Effect Model	SMD (95% CI)	*p*-Value
Culture Type	General Yeast Cultures	16	88.2	Random	2.03 (1.83, 2.22)	<0.0001
	Saccharomyces cerevisiae-Based	8	86.9	Random	1.49 (1.35, 1.64)	<0.0001
	Specialized Yeast Components	2	93	Random	1.45 (0.96, 1.94)	<0.0001
	Composite Yeast Cultures	2	89.4	Random	0.67 (0.09, 1.16)	0.007
Dosage (g/day)	10–50	14	89.9	Random	1.87 (1.67, 2.08)	<0.0001
	60–120	7	72.4	Random	1.47 (1.33, 1.62)	<0.0001
	>120	8	92.5	Random	1.69 (1.43, 1.96)	<0.0001
Feeding Duration (days)	21–30	11	90	Random	1.60 (1.46, 1.74)	<0.0001
	42–56	14	86.3	Random	1.73 (1.52, 1.93)	<0.0001
	60–90	4	84	Random	1.45 (1.07, 1.83)	<0.0001

**Table 3:** Subgroup meta-analysis by culture type, dosage, and feeding duration on milk fat.

Category	Subgroup	Studies (*n*)	*I* ^2^	Effect Model	SMD (95% CI)	*p*-Value
Culture Type	General Yeast Cultures	16	67.90%	Random	0.53 (0.37, 0.69)	<0.0001
	Saccharomyces cerevisiae-Based	6	91.70%	Random	0.63 (0.40, 0.85)	<0.0001
	Specialized Yeast Components	2	94.80%	Random	0.57 (0.16, 0.97)	0.006
	Composite Yeast Cultures	1	0	Random	−0.08 (−0.70, 0.54)	0.806
Dosage (g/day)	10–50	14	87.40%	Random	0.37 (0.21, 0.54)	<0.0001
	60–120	6	54.00%	Random	1.02 (0.78, 1.27)	<0.0001
	>120	6	71.80%	Random	0.4 (0.12, 0.67)	0.005
Feeding Duration (days)	21–30	9	82.80%	Random	0.68 (0.49, 0.87)	<0.0001
	42–56	10	84.90%	Random	0.43 (0.22, 0.63)	<0.0001
	60–90	7	83.90%	Random	0.46 (0.21, 0.71)	<0.0001

**Table 4:** Subgroup analysis of yeast culture characteristics on milk protein percentage.

Category	Subgroup	Studies (*n*)	*I*^2^ (%)	Effect	SMD (95% CI)	*p*-Value
				Model		
Culture Type	General Yeast Cultures	15	87.1	Random	0.88 (0.70, 1.05)	<0.0001
	Saccharomyces cerevisiae-Based	6	78.1	Random	0.96 (0.70, 1.21)	<0.0001
	Specialized Yeast Components	2	94.3	Random	1.43 (0.92, 1.94)	<0.0001
	Composite Yeast Cultures	5	89.2	Random	1.73 (1.23, 2.23)	<0.0001
Dosage (g/day)	10–50	13	87.3	Random	1.15 (0.94,1.36)	<0.0001
	60–120	8	88.3	Random	0.82 (0.56, 1.08)	<0.0001
	>120	8	87.4	Random	0.95 (0.71, 1.18)	<0.0001
Feeding Duration (days)	21–30	8	82.8	Random	1.21 (0.90, 1.34)	<0.0001
	42–56	15	84.9	Random	0.95 (0.75, 1.15)	<0.0001
	60–90	6	83.9	Random	0.86 (0.55, 1.17)	<0.0001

**Table 5:** Subgroup analysis of YC effects on lactose production in dairy cows.

Category	Subgroup	Studies (*n*)	*I*^2^ (%)	Effect Model	SMD (95% CI)	*p*-Value
Culture Type	General Yeast Cultures	14	53.3	Random	0.79 (0.59, 1.00)	<0.0001
	Saccharomyces cerevisiae-Based	6	84.6	Random	0.50 (0.25, 0.74)	<0.0001
	Specialized Yeast Components	2	29	Random	0.56 (0.14, 0.98)	<0.0001
	Composite Yeast Cultures	5	60.1	Random	−0.13 (−0.51, 0.25)	<0.0001
Dosage (g/day)	10–50	13	27	Random	0.43 (0.22, 0.65)	<0.0001
	60–120	8	74.1	Random	0.63 (0.39, 0.87)	<0.0001
	>120	7	86.4	Random	0.66 (0.39, 0.93)	<0.0001
Feeding Duration (days)	21–30	10	44	Random	0.06 (−0.15, 0.27)	<0.0001
	42–56	14	40.5	Random	1.15 (0.94, 1.37)	<0.0001
	60–90	4	55.10%	Random	0.39 (0.06, 0.72)	<0.0001

## Data Availability

No new data were created or analyzed in this study. Data sharing is not applicable to this article.
